# Community engagement and social participation in dengue prevention: A cross‐sectional study in Dhaka City

**DOI:** 10.1002/hsr2.2022

**Published:** 2024-04-02

**Authors:** Md Jubayer Hossain, Manisha Das, Md Wahidul Islam, Muhibullah Shahjahan, Jannatul Ferdous

**Affiliations:** ^1^ Population Health Studies Division, Center for Health Innovation Research, Action, and Learning—Bangladesh (CHIRAL Bangladesh) Dhaka Bangladesh; ^2^ Dhaka Medical College and Hospital Dhaka Bangladesh; ^3^ Department of Microbiology Jagannath University Dhaka Bangladesh

**Keywords:** awareness campaigns, community engagement, cross‐sectional study, demographics, dengue prevention, Dhaka City, religious leaders, urban health

## Abstract

**Background:**

Dengue is a major public health concern in Bangladesh. This study aimed to assess the perceptions and practices of community members in Dhaka regarding community engagement and social participation for dengue prevention.

**Methods:**

A cross‐sectional online survey was conducted in Dhaka City from May 2022 to December 2022. The respondents were randomly selected. The association between community participation and prevention practices was tested using the *χ* ^2^ test.

**Results:**

The findings of this study indicate that the majority of participants (92%) believed that community effort would be relied upon in the event of a dengue outbreak. Environmental cleaning campaigns were the preferred approach, and religious leaders viewed them positively. This study also revealed significant variations in knowledge levels, with those involved in community efforts and mass gatherings demonstrating greater knowledge. This study sheds light on the demographic factors that influence dengue knowledge and provides valuable insights into the development of targeted public health interventions.

**Conclusion:**

The study revealed negative perceptions and limited participation in dengue prevention among participants, with the majority demonstrating a poor understanding of preventive measures. While some showed positive attitudes towards community engagement, significant disparities existed in participation, highlighting the need for targeted educational campaigns and enhanced community mobilization efforts. Moreover, the importance of multisectoral collaboration is emphasized, underscoring the need for coordinated efforts among health departments, NGOs, religious institutions, and community leaders to effectively combat dengue transmission. Recommendations include ongoing educational initiatives, targeted interventions to promote community involvement, and fostering collaboration across sectors to strengthen dengue prevention efforts and to safeguard public health.

## 1 INTRODUCTION

Dengue is a mosquito‐borne infectious disease that spreads rapidly and has become a major global public health concern.^1^, ^2^, ^3^, ^4^, ^5^ According to the World Health Organization (WHO), dengue is one of the top 10 global health threats.^1^, ^5^ Currently, there is no widely available effective treatment or vaccine for dengue.^1^, ^3^, ^4^, ^6^ Although Sanofi Pasteur has developed a dengue vaccine licensed in 24 countries and introduced into public immunization programs in the Philippines and Brazil, there is still a significant gap in global vaccine coverage.^1^, ^6^, ^7^ Additionally, the genetic diversity of the dengue virus, with its various serotypes and genotypes, poses a significant public health challenge, particularly in cases of secondary and tertiary infections, which increase the risk of disease severity.^1^, ^8^, ^9^, ^10^ Dengue has spread to over 125 countries, resulting in an estimated 400 million infections and 40,000 deaths annually.^1^, ^4^, ^6^ Tropical and subtropical regions are the most affected areas, particularly Southeast Asia and South Asia, which account for 70% of the dengue burden.^1^, ^3^, ^4^, ^6^ The spread of dengue is further exacerbated by factors such as rapid urbanization, population growth, and climate change, which create ideal conditions for viral transmission.^2^, ^6^, ^11^ Efforts to prevention the spread of dengue have been hindered by the lack of effective vaccines and treatments, as well as the challenges posed by the genetic diversity of the virus.^3^, ^4^, ^11^ To address these challenges, there is a need for increased investment in the research and development of effective vaccines and treatments as well as improved surveillance and prevention strategies. These efforts should focus on the most affected regions and vulnerable populations, such as children and pregnant women.

Bangladesh, a nation in South Asia, is home to more than 180 million people and has been affected by dengue fever since the initial outbreak in 2000.^1^, ^12^, ^13^, ^14^, ^15^ In recent years, Bangladesh has experienced a series of significant dengue fever outbreaks owing to a variety of risk factors.^1^, ^15^, ^16^ In the absence of appropriate preventative measures, inadequate healthcare facilities, insufficient outbreak readiness, and a lack of community‐level awareness of dengue fever could contribute to public health catastrophes.^1^ Therefore, it is critical to prioritize preventative strategies and increase public awareness of dengue fever in Bangladesh.

Community engagement and social participation are crucial for dengue prevention efforts.^17^, ^18^ Studies have shown that community participation in habitat reduction for Aedes mosquitoes leads to a significant reduction in larval indices and sustainable behavioral changes over time.^18^ Factors such as knowledge, attitudes, and the role of stakeholders can influence community participation in dengue prevention programs.^17^, ^19^, ^20^, ^21^ However, even educated individuals have low knowledge of prevention, and their attitudes towards prevention are mostly negative.^22^ Health workers should actively engage with the community by providing clear education, conducting home visits, and encouraging participation in preventive activities.^23^ Additionally, community capacity‐building programs and improved health‐seeking behaviors are essential for sustained dengue prevention.^24^ School programs that address vector biology and prevention throughout the year can also contribute to effective preventive efforts.^24^ Thus, this study aimed to determine the perceptions and practices of community people in Dhaka regarding community engagement and social participation in dengue prevention.

## 2 METHODS

### 2.1 Study design and setting

This online‐based cross‐sectional study was conducted in Dhaka City from May 2022 to December 2022. The study included participants who met the following criteria: (i) possession of a Bangladeshi resident card, (ii) Internet users, (iii) residents of Dhaka City, and (iv) those who signed an online consent form. Participants with incomplete records or those who did not provide consent were excluded.

### 2.2 Sample size

The required sample size was calculated using the following equation.

$$n=z^{2}pq/d^{2},$$

where *n* is the number of samples, *z* = 1.96 (95% confidence level), *p* is the prevalence estimate (50% or 0.5); as no study found in Bangladesh, *q* = (1 − *p*), *d* = precision limit or proportion of sampling error (5% or 0.05), So, *n* = 1.962 × 0.5 × (1 − 0.5)/0.052 ~ 384.16. A sample size of 385 respondents was estimated with a 95% confidence level and 5% margin of error. The study respondents were randomly selected.

### 2.3 Data collection tools

A structured questionnaire was developed based on a literature review^25^ and modified according to the social context. The questionnaire consisted of three sections: (i) sociodemographic characteristics, (ii) perceptions of dengue prevention through community participation, and (iii) dengue prevention practices. The questionnaire was prepared in English and translated into Bengali for convenience during the data collection. Team members verified the accuracy and meaning of the translated content. A pilot study was conducted, primarily by randomly selecting 15 respondents.

### 2.4 Data collection procedure

Google Forms was used to create the survey questionnaire. The form included questions relevant to the research objectives, ensuring clarity and simplicity in wording the questions. Before the participants began the survey, written consent was obtained from them, following the provision of a clear and concise online consent form. This consent form outlined the purpose of the study, data usage, confidentiality measures, and participants' right to withdraw at any time. The survey link was shared with potential participants through appropriate channels such as email invitations, social media, and websites. Participants had the option to access the survey at their convenience. Ethical clearance was obtained from the Ethical Review Committee of CHIRAL Bangladesh (Reference no: CHIBAN05NOV2022‐0007). Informed consent was obtained from all participants and data analysis was conducted anonymously. Before the data analysis, each case was assigned unique numerical codes to ensure confidentiality. The data collection procedure followed the ethical guidelines of the University of Helsinki for human research.

### 2.5 Statistical analysis

A total of 328 records were included in the final statistical analysis. Categorical data were presented as frequencies and percentages. Continuous data were presented as mean and standard deviation. A particular question was categorized as yes/no/do not know. The association between community participation and prevention practices was tested using the *χ* ^2^ test. The demographic variables are not only confined to the age, sex, and education level of the respondents but also incorporate income and employment status. Detect real confounding variables and utilize them to understand the factors influencing perception outcomes. This in‐depth specification enhances the transparency and reproducibility of our statistical analysis, thereby upgrading the overall robustness of our study. Statistical significance was set at *p* < 0.05. Statistical analyses were performed using R (version 4.0.2) and R Studio (version 1.3.1056) for Windows.

## 3 RESULTS

A total of 328 participants were included in this study. More than half (54%) of the patients were females. The majority (70%) of the participants were aged <25 years. Most respondents (59%) were undergraduates. Unemployment (77%) exceeded employment (23%). Income status was categorized according to the World Bank earnings per day in the BDT of the respondents. Per day earning less than 220 BDT is low, 220‐3000 BDT is middle, and more than 3000 BDT is considered high. Furthermore, most (45%) participants were unsure about their income status. Additionally, the percentage of participants with a medium income was notably higher than that of those with a high income (Table 1).

**Table 1:** Demographic characteristics of study participants (*N* = 328).

Characteristics	*n* (%)
Age group (years)	
<25	229 (70%)
25–44	79 (24%)
45–59	16 (4.9%)
≥60	4 (1.2%)
Level of education	
No education	5 (1.5%)
Secondary	105 (32%)
Graduate	193 (59%)
Postgraduate	25 (7.6%)
Employment status	
Employed	74 (23%)
Unemployed	254 (77%)
Income	
High	7 (2.1%)
Low	73 (22%)
Medium	101 (31%)
None of them	147 (45%)

The perception of dengue prevention among the participants was investigated, revealing their knowledge of executing this operation through community participation (Table 2). Regarding the assumption of organizing people with a view to eradicating dengue, most participants (54%) expressed a good perception, indicating their unity in vector prevention. Less than half (33%) were negative about the idea, whereas 13% remained neutral. Regarding active participation of community leaders, 42% of the participants were positive with this statement, reflecting their satisfaction with community leaders' actions. On the other hand, less than half of the participants (41%) answered No with leaders' actions, and 16% remained neutral. The participants exhibited diverse views on community efforts toward dengue prevention. While the majority (92%) agreed to rely on community efforts, a few (2.1%) were not positive and 5.8% remained neutral. Spraying insecticides to prevention vectors was a community strategy, with 64% of the participants agreeing that they had experience. However, 28% expressed negative responses to this plan of action, and 8.2% remained neutral.

**Table 2:** Frequencies and percentage distribution of respondents regarding their perception to prevention dengue through community participation (*N* = 328).

Statements	*n* (%)
People are organizing to eradicate dengue	
Yes	177 (54%)
No	109 (33%)
Don't know	42 (13%)
Community leaders are active in preventing of dengue	
Yes	139 (42%)
No	136 (41%)
Don't know	53 (16%)
Dengue can be prevented through community efforts	
Yes	302 (92%)
No	7 (2.1%)
Don't know	19 (5.8%)
Does your community spray insecticides for dengue prevention?	
Yes	210 (64%)
No	91 (28%)
Don't know	27 (8.2%)
Your community is involved in the campaign to clean your living environment	
Yes	94 (29%)
No	198 (60%)
Don't know	36 (11%)
Your community shares information about dengue fever	
Yes	166 (51%)
No	136 (41%)
Don't Know	26 (7.9%)
Your community has a linkage with health department NGOs and other agencies for dengue prevention	
Yes	61 (19%)
No	183 (56%)
Don't know	84 (26%)
Religious leaders play important role in awareness about dengue	
Yes	121 (37%)
No	112 (34%)
Don't know	95 (29%)

Regarding community involvement in arranging campaigns for cleaning the living environment, only 29% of the participants stated a positive view, indicating a lack of awareness regarding area cleaning. Nonetheless, 60% were negative for this notion, and 11% remained neutral. The community shares dengue fever‐related information, and 51% of participants answered yes to this. Conversely, 41% were not positive, and 7.9% remained neutral. A multisectoral approach to dengue prevention at the community level was recognized by only a few participants (19%), and the corresponding community was linked to health department NGOs and other agencies. Surprisingly, a large percentage (56%) answered no, while 26% remained neutral. As Bangladesh is one of the most religious and practicing regions in Asia, religious leaders play a vital role in influencing the masses. In the case of dengue prevention roleplay, a large percentage (37%), responded positively, 34% answered negatively, and 29% remained neutral (Table 2).

The responses regarding community participation and dengue prevention practices reflected their awareness and attitudes towards this crucial aspect of dengue epidemic prevention. Among the total participants, only 41 (12%) in this group participated in the community/NGO sprayed fog. A large proportion of 287 participants (88%) held opposing actions, indicating that they were not involved. Regarding community duty to spray insecticides, 222 participants (68%) provided a positive answer for their execution. Regarding the cleaning of water and regular containers, 290 and 165 participants answered Yes, respectively, denoting good practices. A total of 165 participants (50%) covered or followed the recycling of discarded materials from outside the house, whereas the remaining half (50%) did not. Regarding family participation in clean water containers, 246 (75%) responded positively. Eighty percent of the participants cleaned their containers by draining and brushing. Regarding the habit of using mosquito repellents, 249 participants (76%) answered “Yes,” while only 79 participants (24%) did not use it. Among the respondents who used a repellent, coil, or spray, 55% (*n* = 180) used it both during the daytime and evening. However, 148 participants (45%) did not show a habit of using them (Table 3).

**Table 3:** Frequencies and percentage distribution of respondents regarding community participation and dengue prevention practices (*N* = 328).

Community participation	*n* (%)
Do you participate when the community/NGO/Govt is spraying fog?	
Yes	41 (12%)
No	287 (88%)
Do your communities spray insecticides for dengue prevention?	
Yes	222 (68%)
No	106 (32%)
I clean and brush water containers if any larvae inside	
Yes	290 (88%)
No	38 (12%)
I clean my containers one to three times a week	
Yes	225 (69%)
No	103 (31%)
I always keep water containers in my house closed	
Yes	211 (64%)
No	117 (36%)
I cover or recycle discarded material outside the house	
Yes	165 (50%)
No	163 (50%)
All my family members are responsible for cleaning water container	
Yes	246 (75%)
No	82 (25%)
I clean water containers by draining and brushing	
Yes	263 (80%)
No	65 (20%)
I use mosquito repellent	
Yes	249 (76%)
No	79 (24%)
I use repellent or mosquito coil or mosquito spray in the morning and evening	
Yes	180 (55%)
No	148 (45%)

The distribution of perceptions regarding dengue outbreak prevention among respondents revealed variations in awareness levels and potential for targeted educational interventions. Notably, 122 (37%) participants demonstrated good knowledge, whereas 206 (63%) exhibited poor knowledge. Regarding mass gatherings to prevent dengue, 63% of participants demonstrated good knowledge, whereas only 21% of participants answering No exhibited the same. The observed disparity in this issue between positive, negative, and neutral views was statistically significant (*p* = 0.002). Similarly, there was a significant discrepancy in terms of community leader participation, with 53% of participants answering yes exhibiting good knowledge compared to 30% with a negative answer (*p* = 0.004). Community efforts to prevent the dengue outbreak revealed that the highest proportion (100%) of respondents with good knowledge agreed with it. These differences were statistically significant (*p* < 0.001). Additionally, perceptions about the use of spray by the community varied significantly among respondents, with positive (79%) and negative (15%) participants demonstrating good knowledge (*p* < 0.001). The arrangement of various awareness campaigns and seminars also played a prominent role in raising awareness; nonetheless, 50% of participants who did not follow exhibited good knowledge, while 40% of participants who followed this practice demonstrated the same. Conversely, 66% of participants with negative responses and 12% of participants with positive responses displayed poor knowledge, signifying a statistically significant difference in the multisectoral approach and cooperation with outbreak prevention (*p* < 0.001). However, the role of religious leaders did not exhibit a statistically significant association with level of perception (*p* = 0.2). In conclusion, these findings underscore the salient impact of various demographic characteristics on dengue knowledge among the participants, providing valuable insights for targeted educational interventions and public health initiatives (Table 4).

**Table 4:** Association between dengue community participation and dengue prevention practices (good, poor) (*N* = 328).

Statements	Good, *N* (%) = 122 (37%)	Poor, *N* (%) = 206 (63%)	*p* value
People are organizing to eradicate dengue			**0.002**
Yes	77 (63%)	100 (49%)	
No	26 (21%)	83 (40%)	
Don't know	19 (16%)	23 (11%)	
Community leaders are active in preventing of dengue			**0.004**
Yes	65 (53%)	74 (36%)	
No	37 (30%)	99 (48%)	
Don't know	20 (16%)	33 (16%)	
Dengue can be prevented through community efforts			**<0.001**
Yes	122 (100%)	180 (87%)	
No	0 (0%)	7 (3.4%)	
Don't know	0 (0%)	19 (9.2%)	
Does your community spray insecticides for dengue prevention?			**<0.001**
Yes	96 (79%)	114 (55%)	
No	18 (15%)	73 (35%)	
Don't know	8 (6.6%)	19 (9.2%)	
Your community is involved in the campaign to clean your living environment.			**0.002**
Yes	49 (40%)	45 (22%)	
No	61 (50%)	137 (67%)	
Don't know	12 (9.8%)	24 (12%)	
Your community shares information about dengue fever			**0.004**
Yes	76 (62%)	90 (44%)	
No	40 (33%)	96 (47%)	
Don't Know	6 (4.9%)	20 (9.7%)	
Your community has a linkage with health department NGOs and other agencies for dengue prevention			**<0.001**
Yes	37 (30%)	24 (12%)	
No	47 (39%)	136 (66%)	
Don't know	38 (31%)	46 (22%)	
Religious leaders play important role in awareness about dengue			0.2
Yes	43 (35%)	78 (38%)	
No	37 (30%)	75 (36%)	
Don't know	42 (34%)	53 (26%)	

## 4 DISCUSSION

The findings of this study offer valuable insights into the dynamics of dengue prevention among the study population. Notably, there was a significant gender disparity among participants, with over half of them being female. This demographic imbalance may have implications for dengue prevention strategies as sex‐specific factors could influence participation and knowledge dissemination. Furthermore, the dominance of young adults (70% under the age of 25 years) highlights the need to tailor prevention efforts to engage this demographic, recognizing their potential as key contributors to community‐based interventions. The prevalence of unemployment among the participants (77%) raises concerns about financial limitations that may hinder their involvement in dengue prevention activities. Additionally, uncertainty regarding income status in 45% of the respondents underscores the importance of considering economic factors in intervention planning.

This study also sheds light on community perceptions, revealing a generally positive attitude toward organizing communities for dengue prevention, these findings coincide with previous research.^26^, ^27^, ^28^, ^29^ This indicates the potential for community mobilization efforts to effectively combat the disease. However, the mixed responses regarding community involvement suggest the need for improved awareness and engagement strategies to ensure active participation. The knowledge levels among participants varied, with a significant portion demonstrating poor knowledge, emphasizing the necessity for targeted educational interventions to bridge this gap. The present findings confirm the results of previous studies in Bangladesh.^30^, ^31^, ^32^, ^33^, ^34^, ^35^ Intriguingly, those who supported community efforts for dengue prevention exhibited higher knowledge levels, underscoring the potential effectiveness of community‐based interventions in enhancing awareness.

Surprisingly, the role of religious leaders did not show a statistically significant association with perception levels, suggesting that their influence on dengue prevention perception may be limited, as in previous studies.^36^ In conclusion, these findings emphasize the importance of aligning dengue prevention strategies with the demographic characteristics of the target population by considering factors such as sex, age, education, and employment status. Moreover, community engagement and awareness building should be central to public health initiatives, with a focus on addressing knowledge gaps and promoting community‐based approaches to dengue prevention. Further research and tailored interventions are warranted to address the identified challenges and enhance the effectiveness of dengue prevention efforts in this context.

This study sought to address a significant public health concern, as dengue fever is a significant health issue in many urban areas, including Dhaka City. This study's cross‐sectional design allows for the collection of data at a single point in time, which provides an understanding of the current state of community engagement and social participation in dengue prevention. By focusing on Dhaka City, this study offers a detailed examination of a specific urban area, which can aid in tailoring prevention and intervention strategies to the unique characteristics of this location. Additionally, this study's emphasis on community engagement and social participation highlights the importance of involving local communities in disease prevention efforts. The findings of this study could inform public health policies and strategies for dengue prevention in Dhaka City, and potentially in other similar urban settings.

This study has several limitations that should be considered when interpreting the findings. First, the sample representation may be limited, potentially missing hard‐to‐reach or diverse populations. Self‐reporting bias could affect data accuracy because of participants' tendencies to provide socially desirable answers. The cross‐sectional design of this study prevented us from establishing causal relationships or tracking the changes over time. Given the narrow geographic focus of this study, these findings may not be universally applicable. Language and cultural barriers may have influenced participants' responses. The questionnaire design might not have captured the full spectrum of relevant factors. A low response rate or differences in nonparticipant attitudes could introduce selection bias. Furthermore, this study's analysis of the influence of religious leaders may be limited. Finally, temporal and broader contextual factors have not been explored extensively.

We propose several recommendations to enhance dengue prevention in the study population. First, targeted educational campaigns should be developed to address the specific knowledge gaps and demographic characteristics identified in this study, focusing on young adults, females, and individuals with varying education levels. Community mobilization efforts should be promoted to actively engage residents in preventive activities, with an emphasis on involving community leaders and organizations. Socioeconomic support measures are essential for addressing unemployment and income uncertainty and ensuring broader participation. Continuous monitoring and longitudinal studies should be conducted to track changes in knowledge, perceptions, and behaviors over time. Culturally sensitive communication strategies should be employed considering language barriers and cultural factors. Multisectoral collaboration should be encouraged, and opportunities for collaboration with religious leaders should be explored. Behavior‐change interventions should prioritize practical actions, such as proper container maintenance and mosquito repellent use, and awareness campaigns on recycling and environmental cleanup should be launched. Regular assessment of program effectiveness, integration with healthcare services, and local adaptation of strategies are vital for sustained dengue prevention. These recommendations can guide efforts to reduce dengue transmission and improve community engagement with preventive measures.

## 5 CONCLUSIONS

The findings of this study provide valuable insights into perceptions and practices regarding dengue prevention among participants. Despite efforts to raise awareness and implement preventive measures, notable gaps exist in knowledge and participation within the community.

Most participants exhibited negative perceptions regarding dengue prevention, indicating the need for targeted educational interventions to improve their understanding of the disease and its prevention strategies. This underscores the importance of implementing comprehensive public health campaigns to increase awareness and promote proactive measures to combat dengue transmission.

Furthermore, this study highlighted the significant role of community engagement in dengue prevention. While some participants demonstrated positive attitudes towards community participation, there were also notable discrepancies, particularly regarding involvement in activities such as spraying insecticides and organizing cleanliness campaigns. Addressing these disparities and enhancing community involvement through effective communication and mobilization efforts are crucial for fostering a collaborative approach to dengue prevention.

Moreover, this study underscores the importance of multisectoral collaboration in outbreak prevention. Although awareness campaigns and seminars have been instrumental in raising awareness, there is a need for greater cooperation among various sectors, including health departments, NGOs, religious institutions, and community leaders, to effectively address the challenges posed by dengue.

Based on these findings, several recommendations can be made to strengthen dengue prevention. First, there is a need for ongoing educational campaigns targeting communities to improve the knowledge and awareness of dengue prevention measures. Additionally, efforts should be made to enhance community participation through targeted interventions such as training programs and outreach activities. Furthermore, fostering multisectoral collaboration and coordination can help streamline efforts and maximize resources for effective dengue prevention.

In conclusion, addressing the gaps identified in this study through targeted interventions and collaborative efforts is key to reducing the burden of dengue and safeguarding public health in the community. By implementing these recommendations, stakeholders can work towards building a resilient and proactive approach to dengue prevention, ultimately contributing to the well‐being of individuals and communities.

## AUTHOR CONTRIBUTIONS

**Md Jubayer Hossain**: Conceptualization; investigation; writing—original draft; visualization; writing—review and editing; validation; methodology; software; formal analysis; project administration; data curation; supervision; resources. **Manisha Das**: Investigation; writing—original draft; writing—review and editing; validation; data curation; project administration. **Md Wahidul Islam**: Writing—original draft; writing—review and editing; software; validation; investigation. **Muhibullah Shahjahan**: Investigation; validation; writing—original draft; writing—review and editing; software. **Jannatul Ferdous**: Investigation; validation; writing—original draft; writing—review and editing; software.

## CONFLICT OF INTEREST STATEMENT

The authors declare no conflict of interest.

## TRANSPARENCY STATEMENT

The lead author Md Jubayer Hossain affirms that this manuscript is an honest, accurate, and transparent account of the study being reported; that no important aspects of the study have been omitted; and that any discrepancies from the study as planned (and, if relevant, registered) have been explained.

## Data Availability

The data that support the findings of this study are openly available in Dengue_Community_Engagement at https://github.com/chiralbd/Dengue_Community_Engagement.
